# A Systematic Review of the Neuroprotective Effects of Vascular Endothelial Growth Factor (VEGF) in Diabetic Retinopathy and Diabetic Macular Edema: Unraveling the Molecular Mechanisms and Clinical Implications

**DOI:** 10.7759/cureus.51351

**Published:** 2023-12-30

**Authors:** Mansi Yadav, Han Grezenko, Venkata Madusudana Rao Kanukollu, Abdur Rehman, Syed Faqeer Hussain Bokhari, Taufiqa Reza, Carlos D Franco, Srikar P Chilla, Hira Fatima, Jinal Choudhari, Noor Abdullah Yahya, Maaz Amir, Syed Naveed Mohsin

**Affiliations:** 1 Internal Medicine, Pandit Bhagwat Dayal Sharma Post Graduate Institute of Medical Sciences, Rohtak, IND; 2 Translational Neuroscience, Barrow Neurological Institute, Phoenix, USA; 3 Ophthalmology, Stoke Mandeville Hospital, Aylesbury, GBR; 4 Surgery, Mayo Hospital, Lahore, PAK; 5 Surgery, King Edward Medical University, Lahore, PAK; 6 Medicine, Avalon University School of Medicine, Youngstown, USA; 7 Medicine, Universidad Laica Eloy Alfaro, Manta, ECU; 8 Medicine, Care Hospitals, Hyderabad, IND; 9 Epidemiology and Public Health, School of Health Sciences, University of East London, London, GBR; 10 Internal Medicine, Rawalpindi Medical University, Rawalpindi, PAK; 11 Research and Academic Affairs, Larkin Community Hospital, Miami, USA; 12 Medicine, Dubai Medical College, Dubai, ARE; 13 Internal Medicine, King Edward Medical University, Lahore, PAK; 14 Orthopedics, St James Hospital, Dublin, IRL; 15 General Surgery, Cavan General Hospital, Cavan, IRL

**Keywords:** vascular endothelial growth factor, therapeutic strategies, systematic review, clinical implications, molecular mechanisms, retinal ganglion cells, angiogenesis, neuroprotection, vegf, diabetic retinopathy

## Abstract

Diabetic retinopathy (DR) is a leading cause of global visual impairment, necessitating a comprehensive understanding of its vascular and neural components for effective therapeutic interventions. While vascular pathology is well-established, recent evidence suggests a neurodegenerative role in DR. Vascular endothelial growth factor (VEGF), traditionally implicated in angiogenesis, has emerged as a key player with neuroprotective potential. This systematic review evaluates the literature to shed light on molecular mechanisms and clinical implications in this regard. The review adheres to Preferred Reporting Items for Systematic Reviews and Meta-Analyses (PRISMA) guidelines, encompassing a thorough search strategy across multiple databases. Three in vitro studies met the inclusion criteria, highlighting the limited research in this evolving field. Findings suggest VEGF's neuroprotective effects on retinal ganglion cells (RGCs) and retinal neurons, unveiling potential therapeutic avenues. However, concerns arise regarding anti-VEGF therapies' impact on RGC survival. The review discusses the need for further research to delineate specific isoforms and signaling pathways responsible for VEGF-mediated neuroprotection. The delicate balance between angiogenesis and neuroprotection poses challenges in therapeutic development, emphasizing the importance of targeted interventions. Despite limitations, this review provides valuable insights into the intricate relationship between VEGF and neuroprotection in DR, paving the way for future investigations and redefining therapeutic strategies.

## Introduction and background

Diabetic retinopathy (DR), a debilitating complication of diabetes mellitus, stands as a leading cause of visual impairment and blindness globally [1]. As the prevalence of diabetes continues to rise, the burden of DR becomes increasingly significant. Beyond the well-established vascular pathology characteristic of DR, emerging evidence suggests a pivotal role for neurodegeneration in the disease process [2, 3]. The intricate interplay between vascular and neural components in the retina necessitates a holistic understanding to devise effective therapeutic strategies. Among the various molecular players implicated in DR, vascular endothelial growth factor (VEGF) has garnered substantial attention. Historically recognized for its role in angiogenesis, VEGF is now implicated in mediating neuroprotective effects within the diabetic retina [4]. This systematic review aims to comprehensively evaluate the literature surrounding VEGF-mediated neuroprotection in DR, shedding light on the intricate mechanisms and potential clinical implications of this multifaceted phenomenon.

Diabetic retinopathy is characterized by microvascular changes, including capillary non-perfusion, microaneurysms, and neovascularization [5]. While these vascular alterations contribute to vision loss, recent studies underscore the involvement of neurodegenerative processes in the early stages of DR. Retinal ganglion cell dysfunction and loss, along with alterations in other retinal neurons, contribute to the complexity of the disease beyond vascular manifestations [6, 7]. Vascular endothelial growth factor, a key regulator of angiogenesis and vascular permeability, is overexpressed in the diabetic retina. Traditionally viewed as a mediator of pathological neovascularization, recent research suggests a dichotomy in VEGF's role, with evidence pointing towards neuroprotective properties [4, 8, 9]. Understanding this dual role of VEGF is crucial for unraveling the intricacies of DR and devising targeted therapeutic interventions.

Recognizing the importance of neuroprotection in DR, this systematic review focuses on elucidating the role of VEGF in mediating neuroprotection within the context of DR. By critically examining the available evidence, this review seeks to contribute to our understanding of the molecular and cellular mechanisms underlying VEGF-mediated neuroprotection, ultimately paving the way for novel therapeutic avenues. In the subsequent sections, we outline the objectives, methods, and key areas of investigation that will guide our systematic review, offering a structured approach to comprehensively explore the intricate relationship between VEGF and neuroprotection in the context of DR.

## Review

Materials and methods

This systematic review adheres to the established Preferred Reporting Items for Systematic Reviews and Meta-Analyses (PRISMA) guidelines, incorporating a comprehensive approach to identify and assess relevant studies exploring the neuroprotective role of VEGF in DR. The following sections detail the criteria for study inclusion, the search strategy employed, and the methodology for synthesizing the collected data.

Search Strategy

A systematic and thorough search was conducted across multiple electronic databases, including PubMed, Excerpta Medica Database (Embase), and the Cochrane Library, to identify relevant articles. The search strategy involved a combination of medical subject headings (MeSH) terms and keywords related to diabetic retinopathy, VEGF, and neuroprotection. Boolean operators (AND, OR) were used to refine the search and identify studies that met the predetermined inclusion criteria.

Eligibility Criteria

To ensure the selection of high-quality and pertinent studies, specific inclusion criteria were predefined. Studies eligible for inclusion encompassed those investigating the role of VEGF in neuroprotection in the context of diabetic retinopathy. Only articles published in peer-reviewed journals were considered, and a time frame from the inception of relevant databases until October 2023 was designated. Studies focusing on other interventions or lacking sufficient data on neuroprotection were excluded. Studies on only animal cells were also excluded. Only studies that were in the English language and for which the full text was available were included. Gray literature was not considered eligible for this study.

Data Extraction and Synthesis

Two independent reviewers conducted the initial screening of titles and abstracts to identify potentially eligible studies. Subsequently, full-text articles were retrieved and further evaluated for adherence to the inclusion criteria. Any discrepancies between the reviewers were resolved through discussion and, if necessary, in consultation with a third reviewer. Relevant data, including study design, patient characteristics, interventions, and outcomes related to neuroprotection, were systematically extracted using a predefined data extraction form.

Data Analysis

A narrative synthesis approach was employed to summarize the findings from the included studies due to the anticipated heterogeneity in study designs and outcome measures. Key themes and patterns related to the neuroprotective effects of VEGF in DR were identified and presented.

This rigorous methodology ensures the systematic and transparent evaluation of the existing literature, providing a solid foundation for the synthesis of evidence regarding the neuroprotective role of VEGF therapy in DR.

Results

Study Selection Process

**Figure 1:**
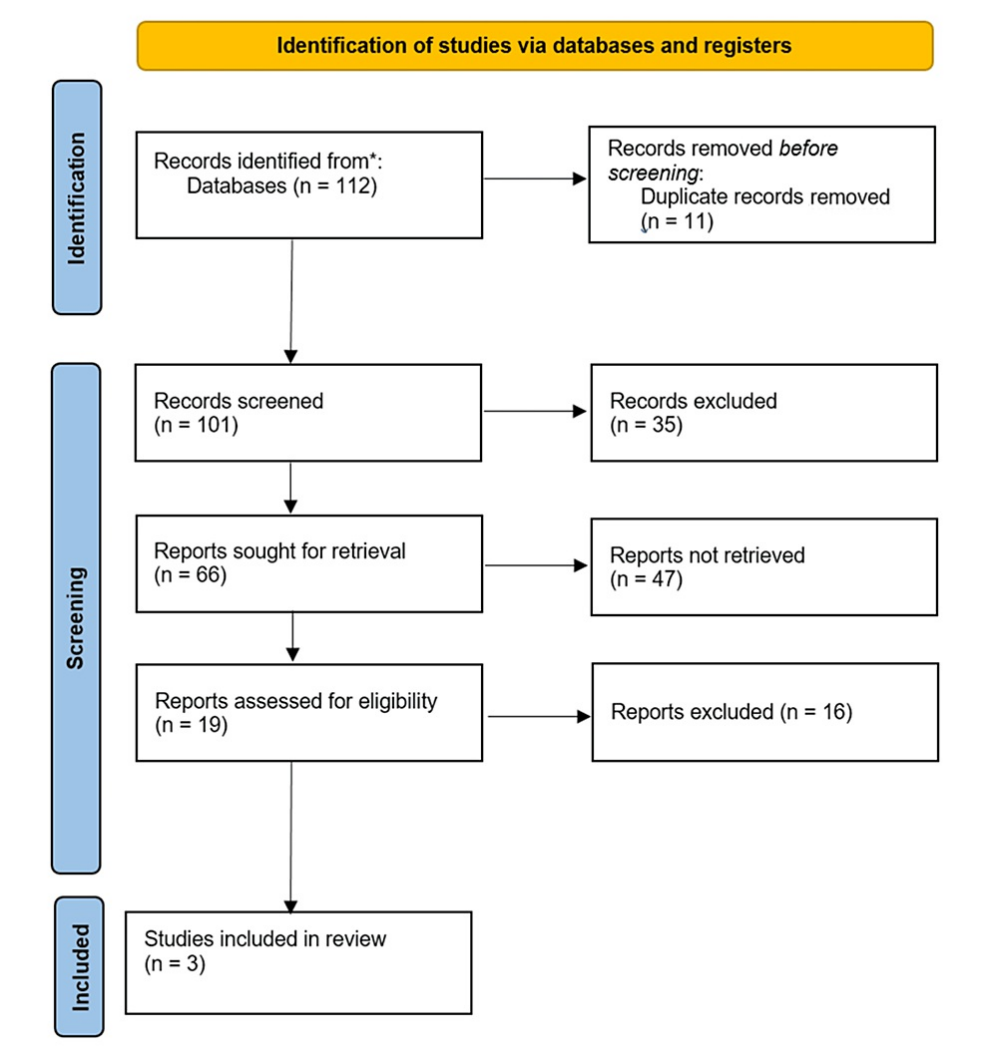
A PRISMA flow diagram of the selection of studies for inclusion in the systematic review PRISMA: Preferred Reporting Items for Systematic Reviews and Meta-Analyses

After conducting four database searches, 112 articles were initially identified. Following the elimination of 11 duplicates, the titles and abstracts of the remaining 101 publications were evaluated. Subsequently, 19 potential studies underwent eligibility verification through a thorough examination of their full texts. Ultimately, three articles satisfied the inclusion criteria. No additional studies meeting the eligibility criteria were found during the examination of the references in the selected articles. The entire process is depicted in the PRISMA flowchart (Figure 1).

Characteristics of the Selected Studies

Overall, three papers met the inclusion criteria. All the studies were in vitro cell culture studies. The studies were one each from France, China, and the United States. The main findings and characteristics of the included studies are mentioned in the following table (Table 1).

**Table 1:** A summary of the studies included in this systematic review

Author	Year	Country	Study type	Drugs studied	Instruments and techniques	Main findings
Froger et al. [10]	2020	France	In vitro cell culture	Anti-vascular endothelial growth factor (VEGF) antibodies: anti-VEGF-A (pan antibody), anti-VEGF-A164 (polyclonal rabbit antibody); ranibizumab control antibody: anti-NF200 (polyclonal rabbit IgG); recombinant proteins: recombinant VEGF-A164, recombinant VEGF-B167	Primary retinal ganglion cell (RGC) culture from rat pups, treatment with drugs and antibodies (anti-VEGF antibodies, ranibizumab, control antibodies, and recombinant VEGF isoforms), immunohistochemistry for protein visualization, Western blotting for protein quantification, cell viability assays (such as MTT (3-(4,5-dimethylthiazol-2-yl)-2,5-diphenyltetrazolium) assay) for quantitative assessment of RGC viability, and statistical analysis to determine the significance of observed effects	The study investigated the neuroprotective role of VEGF therapy in diabetic retinopathy, focusing on RGCs. The results demonstrated that VEGF serves as an autocrine neuroprotective factor for injured RGCs, promoting their survival in vitro. The study identified microglial cells as a source of VEGF synthesis in the retina. Additionally, conditioned medium from mesenchymal stem cells (MSCs), which contained VEGF, also exhibited neuroprotective effects on RGCs. The autocrine release of VEGF by RGCs was confirmed, and the study revealed a correlation between VEGF concentration and RGC survival. Furthermore, experiments with VEGF-trapping molecules and receptor analysis indicated that VEGF-elicited RGC survival involves the VEGF-R1 pathway. The findings also raised concerns about the potential negative impact of anti-VEGF therapies on RGC survival, as evidenced by a significant reduction in retinal nerve fiber layer (RNFL) thickness in glaucomatous patients receiving anti-VEGF treatment for comorbid conditions like age-related macular degeneration (AMD) or diabetic macular edema (DME). These results suggest that VEGF plays a crucial role in the neuroprotection of RGCs and highlight the need for further investigation into the potential consequences of anti-VEGF therapies in patients with compromised RGCs.
Nishijima et al. [11]	2007	United States	In vitro cell culture	VEGFs: VEGF120, VEGF164, PlGF-1, VEGF-E inducible nitric-oxide synthase (iNOS) inhibitor 1400W anti-VEGF antibody, VEGFR1/Fc pegaptanib sodium soluble human VEGF receptor 1 (shVEGFR1), goat polyclonal anti-mouse VEGF-A neutralizing antibody	Intravitreal injections of substances such as VEGF isoforms, growth factors, and inhibitors, Terminal deoxynucleotidyl transferase dUTP nick end labeling (TUNEL) staining for apoptosis detection, immunohistochemical analysis for protein visualization, histological evaluation of retinal tissues, reverse transcription-polymerase chain reaction (RT-PCR) test for gene expression analysis, enzyme-linked immunosorbent assay (ELISA) for VEGF level determination, assessment of volumetric blood flow, retinal explant culture for direct neuroprotective effects evaluation, systemic and local VEGF blockade using soluble human VEGFR1, neutralizing anti-VEGF antibodies, and pegaptanib sodium, retrograde fluorogold labeling for viable retinal ganglion cell count, assessment of p-Akt in mouse retinas, and statistical analysis using Student's t-test and ANOVA.	The study revealed significant findings regarding the neuroprotective role of VEGF-A therapy in diabetic retinopathy. Administering exogenous VEGF-A, specifically the VEGF120 and VEGF164 isoforms, demonstrated a dose-dependent neuroprotective effect, reducing apoptosis in the ganglion cell layer (GCL) and inner nuclear layer (INL) following retinal ischemia. The VEGF-A treatment attenuated ischemia-induced retinal damage, particularly with the VEGF120 isoform, indicating a potential therapeutic avenue. The involvement of VEGF receptor 2 (VEGFR2) in neuroprotection was highlighted, as VEGF-E, a VEGFR2 agonist, exhibited similar effects. The study further emphasized the direct neuroprotective role of VEGF-A on retinal neurons, independent of blood flow changes. Additionally, it explored the endogenous neuroprotective potential of VEGF-A in ischemic preconditioning, unveiling its role in the adaptive response to ischemia. These findings collectively underscore the promising neuroprotective effects of VEGF-A therapy in mitigating retinal ischemic injury associated with diabetic retinopathy.
Zhang et al. [12]	2016	China	In vitro cell culture	Epigallocatechin-3-gallate (EGCG)	In vitro cell culture, where a human retinal endothelial cell (HREC) line was employed. The cells were treated with varying concentrations of EGCG under different glucose conditions. Cell viability was assessed using the MTT assay, providing quantitative information on cell health. Flow cytometry was employed for cell cycle analysis, allowing the determination of cell distribution in different phases. Apoptosis analysis was conducted using flow cytometry with annexin V and propidium iodide (PI) probes. Western blot analysis was utilized to examine the expression levels of proteins associated with the MAPK/ERK-VEGF pathway, including p38 MAPK, phospho-p38 MAPK, ERK1/2, phospho-ERK1/2, and VEGF. An ELISA test was employed to measure the levels of inflammatory markers such as tumor necrosis factor-a (TNF-a), interleukin-6 (IL-6), and intercellular cell adhesion molecule-1 (ICAM-1) in the cell culture supernatant.	The EGCG treatment exhibited protective effects on HRECs under high glucose conditions. The protective mechanisms involved inhibition of apoptosis, modulation of the cell cycle distribution, and suppression of the MAPK/ERK-VEGF pathway. Notably, the study indicates a significant decrease in the expression of VEGF, which is a crucial growth factor associated with diabetic retinopathy (DR) progression.

Discussion

Historically recognized for its involvement in angiogenesis and vascular permeability, VEGF has been a focal point in understanding the vascular pathology of DR [13]. However, recent studies challenge the traditional view, revealing a dual role for VEGF in the context of neuroprotection. While its overexpression is linked to pathological neovascularization, there is growing evidence supporting its neuroprotective properties [14]. The intricate balance between its angiogenic and neuroprotective functions is crucial in deciphering the complexity of DR. This duality raises intriguing questions about the specific isoforms or downstream signaling pathways of VEGF responsible for neuroprotection and whether targeting these pathways could yield therapeutic benefits.

The molecular mechanisms orchestrating VEGF-mediated neuroprotection in DR warrant careful examination. Vascular endothelial growth factor's neuroprotective effects may be linked to its ability to modulate apoptosis, inflammation, and oxidative stress [15]. Experimental evidence suggests that VEGF, through its receptors, may activate intracellular signaling cascades that promote neuronal survival. Additionally, its interaction with other growth factors and cytokines within the diabetic retina could contribute to the observed neuroprotection. However, further research is needed to dissect the specific molecular pathways involved and their relative contributions to neuroprotection [16]. Insights into these mechanisms could pave the way for targeted interventions aimed at preserving retinal neuronal function in DR.

Understanding the neuroprotective role of VEGF has significant clinical implications. As current therapeutic strategies for DR primarily target vascular abnormalities, harnessing VEGF's neuroprotective potential could open new avenues for intervention [17]. The development of therapies specifically designed to enhance VEGF's neuroprotective effects may complement existing approaches, providing a more comprehensive solution to the multifaceted nature of DR. Moreover, identifying patients who may benefit most from such targeted interventions based on their VEGF expression profiles or other biomarkers could optimize treatment outcomes [18]. However, caution is warranted, considering the delicate balance between angiogenesis and neuroprotection. Striking the right balance in therapeutic interventions to ensure neuroprotection without exacerbating vascular complications remains a critical challenge.

Limitations and future directions

While this systematic review provides valuable insights, it is essential to acknowledge its limitations. The limited number of eligible studies highlights the nascent stage of research in this area, emphasizing the need for more robust investigations [19]. Additionally, the heterogeneity in study designs and outcome measures poses challenges in drawing definitive conclusions. Future research should aim to standardize methodologies and outcome assessments to facilitate more comprehensive meta-analyses. Longitudinal studies exploring the temporal dynamics of VEGF expression and its correlation with neurodegenerative changes will enhance our understanding of the evolving role of VEGF in DR.

## Conclusions

This systematic review explores the emerging paradigm of VEGF-mediated neuroprotection in diabetic retinopathy. The dual role of VEGF, molecular mechanisms, and clinical implications discussed herein underscore the complexity of the interplay between vascular and neural components in DR. While the limited number of studies highlights the need for further research, the findings pave the way for future investigations aimed at unraveling the specific isoforms and signaling pathways responsible for VEGF-mediated neuroprotection. Harnessing these insights holds the potential to redefine therapeutic strategies for DR, offering a more holistic approach that addresses both vascular and neurodegenerative aspects of the disease.
